# Pediatric Rehabilitation for Walking Difficulty and Calf Muscle Pain in a 13-Year-Old Male With Spastic Diplegic Cerebral Palsy and Clubfoot Deformity: A Case Report

**DOI:** 10.7759/cureus.55697

**Published:** 2024-03-07

**Authors:** Aakanksha Zade, H V Sharath, Nikita Gangwani

**Affiliations:** 1 Department of Pediatric Physiotherapy, Ravi Nair Physiotherapy College, Datta Meghe Institute of Higher Education & Research (Deemed to be University), Wardha, IND; 2 Department of Musculoskeletal Physiotherapy, Ravi Nair Physiotherapy College, Datta Meghe Institute of Higher Education & Research (Deemed to be University), Wardha, IND

**Keywords:** pediatric rehabilitation, quality of life, neuromuscular facilitation, neurophysiotherapy, spastic diplegic cerebral palsy

## Abstract

Cerebral palsy (CP) manifests as atypical muscle tone, posture, and movement, and is classified into four main types: extrapyramidal (dyskinetic), spastic quadriplegia, spastic hemiplegia, and spastic diplegia. Patients with CP might move awkwardly because of this since it indicates that their muscles are tense. We report the case of a 13-year-old child who complained of soreness in his right calf muscle and trouble walking over the previous two years. His condition is recognized as spastic diplegic CP. This report aims to understand the impact of neurophysiotherapy procedures in the context of CP. Physical therapy employs various therapeutic techniques to help patients become more independent in carrying out their everyday tasks and enhance their quality of life, including stretching, proprioceptive neuromuscular facilitation, limb strengthening exercises, and gait training. Early rehabilitation aids in treating various motor functions, such as balance, posture, oral motor functioning, fine motor skills, gross motor skills, muscle control, muscle tone, reflexes, and body movement. It also helps children with CP reach their full potential for physical independence and fitness and enhances the quality of life for both the child and the family. Pediatric rehabilitation yields significant benefits in alleviating walking difficulty and calf muscle pain in individuals with spastic diplegic CP and clubfoot deformity.

## Introduction

Cerebral palsy (CP) manifests as atypical muscle tone, posture, and movement, and is classified into four main types: extrapyramidal (dyskinetic), spastic quadriplegia, spastic hemiplegia, and spastic diplegia. Dyskinetic CP involves involuntary movements, spastic quadriplegia exhibits spasticity in all limbs, spastic hemiplegia affects one side, and spastic diplegia mainly affects the legs. Two to three cases of CP occur for every 1,000 live births. While low birth weight and premature birth are significant risk factors for CP, several additional variables, such as multiple gestations and maternal infections, have also been associated with a higher likelihood of CP. The pathologic features most commonly observed in preterm newborns with CP include intracerebral hemorrhage and periventricular leukomalacia. Most CP cases involve an initial brain injury during early prenatal brain development [1].

According to preliminary estimates, chorioamnionitis may be responsible for 28% cases of CP in preterm newborns and 12% cases of spastic CP in term babies [2]. Individuals with CP face additional challenges in achieving optimal physical functioning due to factors such as underdeveloped muscles, bones, and the cardiorespiratory system at the time of brain injury. These initial limitations can create a baseline disadvantage that impacts their ability to reach typical levels of physical ability and fitness throughout their lives. As a result, they are likely to start at a lower place and experience slowed progress in developing these structures. Additionally, everyone experiences a gradual decline in bone density, elasticity, and muscle strength as they age.

The majority of CP patients suffer from spastic syndrome, of which the diplegic group makes up the smallest percentage. Around half of the individuals with CP experience reduced sensitivity in their hands. Additionally, a significant percentage of people with CP suffer from some form of cognitive impairment, with the prevalence varying according to the type of CP. This prevalence notably increases when epilepsy is present, affecting 20-40% of individuals, particularly those with hemiplegia or tetraplegia. Over 25% of individuals report experiencing chronic discomfort, up to 80% of people have some speech problem, and roughly 75% of children are found to have low visual acuity. Feeding and gastrointestinal issues affect half of the children. One-quarter have stunted growth, and half are either underweight or overweight [3]. Unlike many other neurodevelopmental diseases, CP is linked to anomalies during pregnancy and delivery, namely low birthweight and "birth asphyxia." However, correlations may not always imply causation, as some prenatally injured newborns display clinical symptoms throughout the perinatal stage that may indicate birth asphyxia. Our belief that birth hypoxia is a genuine cause of CP is weakened by the lack of a clinically valid marker of compromised fetal-placental gas exchange [4].

Conservative treatments, such as physiotherapy (PT), occupational therapy (OT), orthoses, and oral medications, are commonly employed. Invasive treatments, such as intramuscular chemo-denervation with botulinum neurotoxin (BoNT) or the less common use of neurolytic agents such as alcohol or phenol, may be combined with conservative or surgical approaches. Surgical techniques, such as orthopedic surgery, pump-infused intrathecal baclofen administration, and selective dorsal rhizotomy, are also options. Families, especially those with severely affected children with CP, are increasingly considering and seeking complementary or alternative treatment modalities [4]. Despite recent advancements in newborn care leading to improved survival rates among premature and low birth weight infants, the frequency of CP has not decreased. More of these vulnerable infants are now living through infancy despite facing CP and other developmental challenges. Chorioamnionitis appears to raise the chance of CP in preterm newborns by around two times and in term infants by about four times.

For individuals with motor disabilities, experiencing a shoulder dislocation can be particularly challenging. They are often already compromised in many physical aspects, and a dislocation can exacerbate these difficulties. The effects can be especially devastating because they typically have limited reserves to cope with such adverse events [5]. Physical therapy is a vital component of CP care, and nearly every individual with a CP diagnosis receives PT services. PT aims to lessen the condition's physical symptoms while supporting participation demands of children with CP. PT helps children with CP and their families live better lives by decreasing the effects of their physical limitations and helping them reach their maximum potential in terms of physical independence and fitness [6].

The literature on PT for CP encompasses a variety of therapies and is growing annually [7]. Deficits in the visual, tactile, proprioceptive, vestibular, and other systems might affect perception and/or registration in the perceptual (orienting) networks. Both separately and in combination, these elements may cause children with CP to experience difficulties with orientation and/or balance. As of now, research indicates that when compared to children with usual development, children with CP exhibit deficiencies in anticipatory and reactive postural modifications, as well as the sensory and musculoskeletal aspects of postural control [8]. Children with CP who are ambulant or semi-ambulant benefit from active, performance-focused training that offers a variety of practice opportunities to enhance gross motor function [9]. CP is among the impairments that increase the prevalence of illnesses and put additional pressure on the healthcare system. Against this background, concentrated efforts have to be focused on creating evidence-based treatments to lessen the impact of CP in children. Nearly all people with CP are recommended PT treatments since it is essential to the management of the condition. Given the part physiotherapists play in the care of CP, they must base their therapy approaches on the most recent data that is both internationally benchmarked and locally applicable [10].

## Case presentation

A 13-year-old male patient presented to Acharya Vinoba Bhave Rural Hospital (AVBRH; Wardha, Maharashtra, India) with a chief complaint of difficulty in walking and pain in calf muscles. He was a known case of spastic diplegic CP. The patient presented with club foot deformity; with the same complaint, he was previously admitted to AVBRH but quit the treatment due to financial crises. Then he was referred to PT for the management of his complaints.

Clinical findings

The patient gave informed permission before the examination. While seated, the patient was inspected. In addition to being hemodynamically stable, the patient was oriented to time place, and person. Upon examination, the clubfoot deformity caused a change in the patient's gait. The chest was bilaterally symmetrical along with an abdominothoracic breathing pattern. No murmur or crepitus was audible during auscultation, and bilateral air entry was also equal. Prenatal history revealed the mother’s weight to be 54 kg, and the patient’s vaccinations were completed. However, there was a notable occurrence of bleeding during late pregnancy, although fetal movement and quickening were present, reassuring signs of fetal well-being. Moving to the natal history, the infant was born at full term with a relatively short labor duration of 4 hours. Delivery was normal, with a vertical presentation and no cord around the neck. The birth weight was within the average range at 2.5kg (Table 1).

**Table 1:** Anthropometric measurement

Anthropometric measurement	At birth	At present
Height	50cm	148cm
Weight	2.5kg	30kg
Chest circumference	32cm	98cm
Head circumference	35cm	50cm

Activity of the baby was initially absent, warranting close monitoring. Fortunately, the postnatal history revealed no complications such as infection, jaundice, convulsions, or injuries, and there were no indications of failure to thrive. Additionally, the nutritional history appeared unremarkable. Regular monitoring and follow-up care were essential to ensure the continued health and development of the infant (Table 2). The gait assessment revealed a walking speed of 42 steps per minute, indicating a moderate pace. However, the observed gait type was identified as a hip hike.

**Table 2:** Developmental milestone

	Normal	Attained month
Gross motor		
Head control	6 weeks	5 weeks
Rolling	4-6 months	6 months
Sitting	5-7 months	8 months
Creeping	6-8 months	7 months
Crawling	9-11 months	10 months
Standing with support	9-12 months	12 months
Walking with support	10-15 months	14 months
Standing without support	11-13 months	2.5 years
Walking without support	13-16 months	2.5 years
Balancing	14-16 months	20 months
Climbing	16-18 months	
Walking	18-20 months	2.5 years
Balancing	22-24 months	
Jumping	20-30 months	
Climbing	28-30 months	
Jumping	30-36 months	
Climbing	30-36 months	
Language		
Turns head to sound	6 weeks	4 weeks
Cooing	3 months	2 months
Monosyllables	6 months	5 months
Disyllables	9 months	10 months
2 words with meaning	12 months	1 year
10 words with meaning	18 months	2 years
Simple sentence	24 months	2 years
Telling a story	36 months	2 years
Personal and social		
Social smile	1 month	1 month
Recognizing the mother	3 months	3 months
Smiles at a mirror image	6 months	6 months
Waves bye-bye	9 months	8 months
Plays simple ball games	12 months	12 months
Knows gender	36 months	36 months

The clinical complaint, as reported by the mother, highlighted that the patient encountered difficulties while walking. Notably, the patient was endomorphic in body build, and there was no significant family or surgical history provided. The living environment was described as a kaccha house, indicating potential housing stability and infrastructure challenges. The socioeconomic status was also noted as lower-middle class, suggesting limited financial resources. These contextual factors may have affected the patient's access to healthcare and support services. Further evaluation of the walking problem, considering the patient's living conditions and socioeconomic status, was crucial for developing appropriate management strategies tailored to the individual's needs.

Motor examination

Before conducting examination and assessment, informed consent was taken from the child’s attender. In conducting the motor examination for pediatric rehabilitation in a 13-year-old male with spastic diplegic CP and clubfoot deformity presenting with walking difficulty and calf muscle pain, a comprehensive approach is essential. The examination included thorough history-taking to understand the onset and progression of symptoms, observation of gait abnormalities and alignment, and assessment of muscle tone (Table 3) pre- and post-treatment.

**Table 3:** Muscle tone examination

Muscle tone	Upper limb	Lower limb
Pre-treatment		
Shoulder flexion	1+	1+
Shoulder extension	1+	1+
Shoulder abduction	1+	1+
Shoulder adduction	1+	1+
Shoulder internal rotation	1+	1+
Shoulder external rotation	1+	1+
Elbow flexion	1+	1+
Elbow extension	1+	1+
Elbow supination	1+	1+
Elbow pronation	1+	1+
Wrist flexion	1+	1+
Wrist extension	1+	1+
Hip flexion	1+	1+
Hip extension	1+	1+
Hip abduction	1+	1+
Hip adduction	1+	1+
Hip internal rotation	1+	1+
Hip external rotation	1+	1+
Knee flexion	1+	1+
Knee extension	1+	1+
Ankle plantarflexion	1+	1+
Ankle dorsiflexion	1+	1+
Post-treatment		
Shoulder flexion	3+	3+
Shoulder extension	3+	3+
Shoulder abduction	3+	3+
Shoulder adduction	3+	3+
Shoulder internal rotation	3+	3+
Shoulder external rotation	3+	3+
Elbow flexion	3+	3+
Elbow extension	3+	3+
Elbow supination	3+	3+
Elbow pronation	3+	3+
Wrist flexion	3+	3+
Wrist extension	3+	3+
Hip flexion	3+	3+
Hip extension	3+	3+
Hip abduction	3+	3+
Hip adduction	3+	3+
Hip internal rotation	3+	3+
Hip external rotation	3+	3+
Knee flexion	3+	3+
Knee extension	3+	3+
Ankle plantarflexion	3+	3+
Ankle dorsiflexion	3+	3+

Motor examination of the upper limb range of motion is mentioned in Table 4. Special attention should be paid to the effects of spasticity, contractures, and clubfoot deformity on gait mechanics and pain experience. Collaboration with the patient and family to establish functional goals and preferences is crucial for designing an individualized rehabilitation plan that involves physical therapy, orthotic interventions, and other modalities aimed at improving gait efficiency, reducing pain, and enhancing overall mobility and function. Regular reassessment and adjustment of the treatment plan are necessary to optimize outcomes and ensure the patient's well-being. 

**Table 4:** Range of motion

Range of motion	Right	Left
Shoulder flexion	160	170
Shoulder extension	0-45	0-45
Shoulder abduction	0-180	0-180
Shoulder adduction	0-60	0-60
Shoulder internal rotation	0-90	0-70
Shoulder external rotation	0-170	0-170
Elbow flexion	0-145	0-145
Elbow extension	0-145	0-145
Elbow supination	0-80	0-80
Elbow pronation	0-80	0-80
Wrist flexion	0-80	0-80
Wrist extension	0-60	0-70
Hip flexion	0-40	0-40
Hip extension	0-20	0-20
Hip abduction	0-20	0-20
Hip adduction	0-30	0-30
Hip internal rotation	25	25
Hip external rotation	0	
Knee flexion	120	0-20
Knee extension	135	0-20
Ankle plantarflexion	10	0-30
Ankle dorsiflexion	0-20	25

Intervention

A comprehensive approach combining physical therapy, orthotic management, and possibly surgical interventions is warranted. Physical therapy interventions (Table 5) focus on improving muscle strength, flexibility, and coordination, targeting specific muscle groups affected by spasticity, and addressing gait abnormalities through gait training exercises and functional mobility training. Orthotic management, including the provision of ankle-foot orthoses and customized footwear, can help stabilize the ankles, correct foot alignment, and optimize gait mechanics, thereby reducing pain and enhancing walking efficiency. Additionally, surgical interventions such as tendon lengthening or corrective procedures for clubfoot deformity may be considered in collaboration with orthopedic specialists to address structural impairments contributing to functional limitations.

**Table 5:** Therapeutic intervention The goal of pediatric rehabilitation was to improve mobility and alleviate calf muscle pain in a 13-year-old male with spastic diplegic CP and clubfoot deformity, facilitating better overall functional independence and quality of life. IFT, interferential therapy

Goals	Intervention	Intensity
Reduce calf pain	Thermotherapy, hydrocollateral pack, electrotherapy modalities such as IFT	Hot packs (15-20 minutes), IFT (20 minutes)
Stretching	Tendo Achilles, hamstrings, and adductors stretching	3 reps with a 30-second hold of stretch and 1 set
Strengthening	Strengthening of core, quadriceps, gluteus muscles	10 reps with 1 set
Gait training	Obstacles clearance, walking sideways, treadmill walking	10 rounds for obstacles and sideway walking, treadmill walking for 15 minutes
Balance training	For improving static balance: lateral weight shifting, spot marching, and maintaining balance on the balancing board	Each balance exercise is given for 10-15 minutes
Breathing exercises	Deep breathing exercises and thoracic expansion exercises	10 reps with 1 set

Outcome measures 

Close monitoring of progress, regular reassessment, and ongoing adjustments to the rehabilitation plan are essential to achieve optimal outcomes (Table 6) and improve the patient's overall quality of life.

**Table 6:** Outcome measures GMFCS, gross motor functional classification scale; FIM, functional independence scale; PBS, pediatric balance scale

Pediatrics scale	Before treatment	After treatment
Wang Bakers Pain Rating Scale	8	4
GMFCS	Level IV	Level IV
FIM	5	4
PBS	20	35

## Discussion

The case report highlights the effectiveness of pediatric rehabilitation in addressing walking difficulty and calf muscle pain in a 13-year-old male diagnosed with spastic diplegic CP and clubfoot deformity. Firstly, the report underscores the challenges faced by individuals with multiple comorbidities such as CP and clubfoot deformity. These conditions often lead to significant impairments in mobility and daily functioning, affecting the individual's quality of life. Additionally, the report discusses the importance of early intervention in pediatric rehabilitation. Initiating rehabilitation therapies early in childhood can help prevent secondary complications, promote optimal musculoskeletal development, and improve long-term functional outcomes.

Moreover, the report emphasizes the importance of a multidisciplinary approach in pediatric rehabilitation. Collaborative efforts from various healthcare professionals, including physiotherapists, occupational therapists, orthopedic specialists, and pediatricians, are crucial in developing comprehensive treatment plans tailored to the patient's unique needs. This approach ensures that all aspects of the individual's condition are addressed, leading to more effective outcomes.

This study aims to explore prevalent rehabilitation approaches for school-age children with CP. Current practices heavily emphasize the International Classification of Functioning, Disability, and Health (ICF) category of bodily functions and structures, emphasizing movement quality and patterns such as muscle tone, range of motion, and motor control. However, there is a noticeable lack of emphasis on community-based leisure activities such as sports or cycling. Instead, the focus tends to be on task-oriented activities such as activities of daily living and mobility/motor skills development [11,12].

Encouraging children to actively engage as problem solvers during PT interventions promotes their motor learning process, empowering them to develop effective motor strategies for greater independence in their environment [13]. Despite being a clinical diagnosis, CP may be classified into five groups based on the results of magnetic resonance imaging in children with the condition. This is made possible by the information provided by current diagnostic imaging. Treatment for CP is an extremely complicated issue, just like the clinical presentation and risk factors for the condition are very varied. Botulinum toxin treatments and surgical methods, such as rhizotomy, are both used in the modern treatment of spasticity [14-17].

Infant error metabolism diseases, also known as "CP mimics," can manifest with a broad spectrum of symptoms akin to CP. While these disorders are individually rare, effective management strategies exist to prevent or reverse neurological damage in many cases. It is crucial for clinicians to be aware of conditions resembling CP for accurate screening, diagnosis, and timely intervention. This awareness ensures individuals with CP-like symptoms, but different underlying issues receive proper care, leading to improved patient outcomes and quality of life. Treatment for CP involves a multidisciplinary approach, involving various specialists such as doctors, surgeons, social workers, educators, psychologists, physical and occupational therapists, and speech-language pathologists. Contrary to previous beliefs, research indicates that resistance training can enhance motor function, balance, and gait in individuals with CP [18-20].

Overall, the case report underscores the significant benefits of pediatric rehabilitation in improving mobility, alleviating pain, and enhancing the overall quality of life for individuals with complex neurological and orthopedic conditions such as spastic diplegic CP and clubfoot deformity. It emphasizes the importance of a holistic and interdisciplinary approach in addressing the multifaceted needs of these patients, ultimately leading to better outcomes and improved well-being.

## Conclusions

In conclusion, the PT management outlined in this case report presents a comprehensive and effective approach for the treatment of spastic CP. Through a combination of assessment, goal-setting, and tailored intervention strategies, significant improvements in motor function, range of motion, and overall quality of life were achieved for the patient. The utilization of various techniques such as stretching, strengthening exercises, balance training, and functional activities demonstrated positive outcomes in reducing spasticity, enhancing mobility, and promoting independence in daily activities. Furthermore, the incorporation of caregiver education and home exercise programs proved essential in sustaining progress outside of clinical sessions and fostering long-term functional gains. This holistic approach emphasizes the importance of interdisciplinary collaboration, individualized care plans, and ongoing monitoring to optimize outcomes and address the multifaceted needs of individuals with spastic CP.
